# Short-Term and Long-Term Effectiveness of Intensive Interdisciplinary Pain Treatment for Children and Adolescents with Chronic Headache: A Longitudinal Observation Study

**DOI:** 10.3390/children8030220

**Published:** 2021-03-12

**Authors:** Meltem Dogan, Boris Zernikow, Julia Wager

**Affiliations:** 1 German Paediatric Pain Centre, Children’s and Adolescents’ Hospital, 45711 Datteln, Germany; b.zernikow@kinderklinik-datteln.de (B.Z.); j.wager@deutsches-kinderschmerzzentrum.de (J.W.); 2 Department of Children’s Pain Therapy and Paediatric Palliative Care, Faculty of Health, School of Medicine, Witten/Herdecke University, 58448 Witten, Germany

**Keywords:** pediatric, chronic pain, headache, intensive interdisciplinary pain treatment

## Abstract

Pediatric chronic headache causes significant impairment to those affected and bears the risk of aggravation into adulthood. Intensive interdisciplinary pain treatment (IIPT) was found to positively affect pain-related and emotional outcomes in pediatric patients with chronic pain up to 4 years after treatment. This study was aimed at investigating the effect of IIPT on solely pediatric chronic headache patients. As part of a longitudinal observation study, *n* = 70 children and adolescents with chronic headache receiving IIPT were included, of which *n* = 47 completed the assessment at four assessment time points: before treatment (PRE) and at three follow-ups (6-MONTH FOLLOW-UP, 1-YEAR FOLLOW-UP, and 4-YEAR FOLLOW-UP). Pain-related (pain intensity, pain-related disability, school absence), as well as psychological outcome domains (anxiety, depression), were investigated. The results support the short-term and long-term efficacy of IIPT for pediatric chronic headache patients regarding the pain-related outcome measures by significant reductions from PRE to all follow-up measure points. For anxiety and depression, only short-term improvements were found. Future studies should specifically focus on the identification of methods to consolidate the beneficial short-term effects of IIPT on psychological outcome domains in the long term.

## 1. Introduction

Severe chronic pain in children and adolescents is a highly impairing health condition, not only for the 5% to 8% of children severely affected [1,2], but also for their families and society as a whole [3,4]. More than one-third of adolescents with chronic pain experience headaches, making this pain location the most prevalent after lower limb pain (41%) [1]. Moreover, children and adolescents with chronic headaches were found to be more likely to experience more severe chronic pain syndromes than those with other pain locations [2]. The majority (60%) of pediatric headache patients report medication overuse and show significantly more symptoms of anxiety, depression, and somatization compared with healthy controls [5].

Findings of numerous studies demonstrate that intensive interdisciplinary pain treatment (IIPT) represents a short-term as well as a long-term effective treatment for pediatric patients with severely disabling chronic pain [6,7]. In this mixed group of various pain complaints, IIPT was found to positively impact pain-related (e.g., pain intensity, pain-related disability, school absence), as well as psychological outcome parameters (e.g., anxiety, depression, catastrophizing) [7,8]. Within IIPT, the joint and interconnected treatment of a variety of disciplines (e.g., pediatricians, psychotherapists, nurses, physiotherapists) [9] is usually delivered in a 3- to 4-week inpatient setting [10]. To address the most relevant aspects and consequences of pediatric chronic pain, IIPT targets the underlying pain mechanisms, focuses on co-morbidities such as anxiety or depression, teaches pain management strategies, and concurrently integrates psychosocial issues such as family functioning and parents’ reactions to pain [10].

However, while numerous studies have investigated the usefulness of IIPT for children with chronic pain and functional disability [6,10], few have focused on the effectiveness of IIPT in chronic headache patients. Benore et al. [11] retrospectively examined the treatment effect at 12-month follow-up for *n* = 135 pediatric patients with chronic headache receiving IIPT between 2008 and 2014. They demonstrated a significant linear improvement in pain-related and psychological parameters, such as daily and family functioning as well as school absence. The drop-out rate in this study was very high (87.4%). In a retrospective study with a convenience sample of *n* = 50 headache patients, Shulman et al. [12] also showed an improvement in pain intensity, pain-related disability, and school attendance 6–8 weeks after receiving IIPT. However, the results of both studies may be biased due to the retrospective nature and high dropout rate of the former, and the use of convenience sampling in the latter.

This prospective longitudinal observational study aims to provide further evidence for the short-term effectiveness of IIPT for pediatric chronic headache patients and to investigate the long-term utility of IIPT for this patient group over 4 years.

## 2. Materials and Methods

### 2.1. Study Design

The study was initially implemented as a larger randomized controlled trial (RCT) with a wait-list control condition. The patients on the waitlist received the same IIPT after a short time delay of 3–4 weeks. Therefore, the group comparison of the RCT was conducted when the intervention group finished their 3–4-week inpatient treatment and the control group was about to start their inpatient treatment. Because after this time point both groups had received the same treatment, the study was then transferred into a longitudinal observation design [8]. Longitudinal data were collected prospectively at four assessment points for all patients as follows: initial assessment at the first appointment at the pain center (PRE) and six months (6-MONTH FOLLOW-UP), one year (1-YEAR FOLLOW-UP), and four years (4-YEARS FOLLOW-UP) after study intake. As the primary randomization does not affect the longitudinal data, the two groups are combined for the follow-up analyses. A sub analysis of RCT data was abandoned due to the small sample size of headache patients in each group, which reduced statistical power. The initial study included a mixed pain sample. Here, we report on a subset of longitudinal data of only the headache sample.

### 2.2. Study Sample

Patients were eligible for the study if they (1) presented at the German Pediatric Pain Centre during the recruitment period (November 2009–July 2011); (2) fulfilled a priori-defined criteria for the admission to IIPT (3) were between 9 and 17 years old; (4) had age-appropriate German language and reading comprehension; and (5) they and their parents agreed to participate in the study. Criteria for admission to IIPT were: (1) no malignant disease, (2) age-appropriate comprehension of German, (3) severe pain-related disability, and (4) motivation for therapy judged by a clinician during the initial appointment. Moreover, three of the following five requirements had to be met: (1) pain duration ≥ six months, (2) average pain intensity of ≥5 over the preceding seven days on a 0 to 10 Numeric Rating Scale (NRS), (3) pain peaks (pain intensity ≥8) occurring more than twice per week, (4) a minimum of one week absent from school due to pain within the preceding four weeks, and (5) high pain disability as perceived by the adolescent (P-PDI ≥ 36) [13]. Study exclusion criteria were (1) the diagnosis of complex regional pain syndrome because of a different treatment approach and (2) previous treatment with IIPT.

Children and adolescents were referred to the pain center by their primary care pediatricians. Study participants were recruited for the study by a researcher at the initial appointment (PRE) at the pain center. For this subsample analysis with chronic headache, a total of *n* = 70 children who reported headache as their main pain location were eligible.

**Figure 1:**
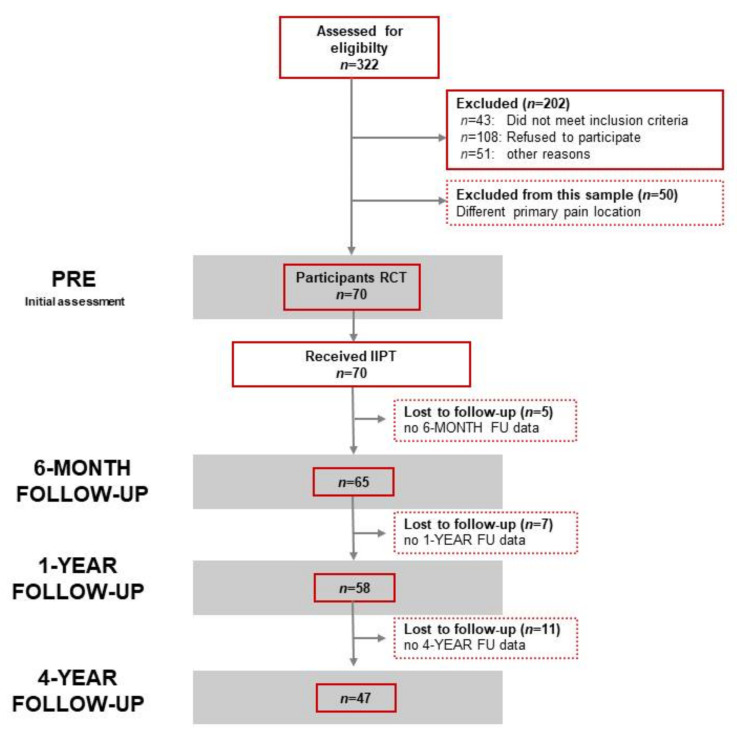
Flowchart for the chronic headache subsample.

Of these, *n* = 65 participated in the 6-MONTH FOLLOW-UP, *n* = 58 in the 1-YEAR FOLLOW-UP, and *n* = 47 participated in the 4-YEAR FOLLOW-UP. The study flowchart for this analysis is depicted in Figure 1.

At admission, no significant differences were found between participants who participated in the follow-ups and those who refused to participate in the follow-ups regarding demographic characteristics (i.e., age, sex), pain parameters (i.e., pain intensity, pain-related disability, pain-related school absence), or psychological characteristics (i.e., anxiety, depression, and catastrophizing; assessed via Fisher’s test and Mann-Whitney U-test).

### 2.3. Procedure

At the initial appointment at the pain center (PRE), the patients completed the study questionnaires. Six months (6-MONTH FOLLOW-UP), as well as one year after the initial appointment (1-YEAR FOLLOW-UP), both study groups received the questionnaires via mail and were asked to return them to the pain center. The long-term follow-up assessment four years after enrollment (4-YEAR FOLLOW-UP) was implemented (1) through a structured 30-min telephone interview by trained student research assistants and (2) additional paper-pencil questionnaires. This procedure differing from the other two assessment points was chosen to inform and remind the patients verbally about the study, to check their mailing address, and to collect item data (primarily core pain characteristics). Furthermore, the phone call aimed to increase the study participation rate. After the interview, the study participants were asked if they were willing to complete further questionnaires containing more complex items regarding psychological outcome domains (see ‘Measures’ for details). These were sent to them via mail. Pain-related parameters were assessed within the interviews, while data on psychological outcome measures were collected via the questionnaires.

### 2.4. Intensive Interdisciplinary Pain Treatment (IIPT)

The IIPT followed a manualized treatment approach [14] provided in a 3- to 4-week inpatient setting by an interdisciplinary team of pediatricians, clinical psychologists, pediatric nurses, physiotherapists, occupational therapists, and social workers [9]. IIPT consisted of six treatment modules: (1) education and realistic goal determination; (2) acquisition of pain-coping strategies; (3) treatment of co-occurring emotional distress; (4) family therapy; (5) optional therapy-related drug treatment or physiotherapy; and (6) relapse prevention [14]. Each treatment week included three to four single sessions with a child psychotherapist, with every session containing special homework (e.g., exercising the newly learned distraction techniques). Moreover, a therapeutic session with the patient’s family and a consultation with a pediatrician was conducted once a week. Beyond this, the pediatrician could be consulted whenever needed. Patients engaged in two group-therapy sessions and various group activities (e.g., physiotherapy, sports, cooking, arts, and music therapy) each week. Overall, patients obtained a total of five to eight hours of therapeutic activities per day [14]. During IIPT, patients’ families were actively involved and provided with pain-related education to dissolve dysfunctional parental cognitions and maladaptive behavior patterns, and to strengthen the parents’ ability to cope with the child’s pain [14]. Pharmacological treatment was encouraged in children with migraines. If current pharmacological migraine treatment was not sufficient, it was further adapted during IIPT. At the end of IIPT, the children and adolescents received a personalized discharge plan with recommendations such as attending outpatient psychotherapy. Three and six months after discharge, patients and families were offered a follow-up meeting with the treating pediatrician and psychotherapist at the pain center.

### 2.5. Measures

#### 2.5.1. Average Pain Intensity

The average pain intensity within the past seven days was reported by the patients on a numerical rating scale (NRS) ranging from 0 to 10 (0 = no pain to 10 = strongest pain). This assessment method is valid and sensitive to change in pediatric samples [15].

#### 2.5.2. Pain-Related School Absence

Patients were asked to report the number of days they had missed school due to pain within the last four weeks (maximum of 20 days).

#### 2.5.3. Pain-Related Disability

Pain-related disability was assessed via the validated Paediatric Pain Disability Inventory (PPDI) [16,17], which consists of 12 self-report items assessing pain-related disability in daily activities (e.g., reading or doing sports) on a 5-point Likert scale (1 = never to 5 = always). The total score ranges from 12 to 60 with higher values indicating greater disability. The tool has demonstrated good internal consistency (in this sample, Cronbach’s α = 0.87).

#### 2.5.4. General Anxiety

General anxiety was measured using the validated Anxiety Questionnaire for Pupils (AFS) [18]. This measure is frequently used in adolescent samples and allows a norm-based comparison with healthy school children. These norms were utilized to transform individual raw scores to T-values with a mean of 50 and a standard deviation of 10. We used the 15-item general anxiety subscale, which has shown good internal consistency (in this sample, Cronbach’s α = 0.70).

#### 2.5.5. Depression

Depression was assessed using the validated Depression Inventory for Children and Adolescents (DIKJ) 2000 [19]. This 26-item questionnaire also allows a norm-based comparison with nonclinical schoolchildren. The scale shows good internal consistency (in this sample, Cronbach’s α = 0.84).

### 2.6. Ethics

The study procedure was approved by the ethics committee of Witten/Herdecke University (Nr. 78/2007). Moreover, it was registered in the “German Clinical Trials Register” (Deutsches Register Klinischer Studien, DRKS; DRKS-ID: DRKS00000337) and the ISRCTN register (trial number ISRCTN91385238).

### 2.7. Data Analysis

All data analyses were carried out with SPSS^®^ software (release 27.0 for Windows^®^). The following statistics were used:

First, the demographic and clinical characteristics of the sample at PRE were described by the use of descriptive statistics (mean (M) and standard deviation (SD) for continuous variables; frequencies and percentages for categorical variables). Equivalence of the group of responders and non-responders at 4-YEAR FOLLOW-UP was established by testing for differences in these variables between the two groups (t-tests for continuous variables; chi-square tests for categorical variables).

For the longitudinal analyses, repeated measures analyses of variance (ANOVA) with four assessment time points (PRE, 6-MONTH FOLLOW-UP, 1-YEAR FOLLOW-UP, and 4-YEAR FOLLOW-UP) were used to examine the influence of time on changes on each of the continuous dependent variables (pain intensity, pain-related disability, school absence, depression, anxiety). The Greenhouse-Geisser adjustment was used to correct for violations of sphericity. In the case of a detected significance, Bonferroni-adjusted post hoc analyses were applied to locate the significant change.

## 3. Results

### 3.1. Demographic and Clinical Characteristics

The total study subsample was composed of *n* = 70 children and adolescents with chronic headaches. Study participants were on average 14.5 years old (SD = 2.11) and 77.1% of them were female. All patients were diagnosed with the main diagnosis of a somatoform or chronic pain disorder (ICD-10 F45.40 or F45.41). Secondary diagnoses were migraine in 41% and tension-type headache in 19% of the sample. The demographic and clinical characteristics at PRE of the total study subsample and the group of participants, as well as dropouts of the subsample at 4-YEAR FOLLOW-UP, are depicted in Table 1. The two groups did not differ significantly in socio-demographic or pain characteristics at study inclusion (see Table 1). For longitudinal analyses, *n* = 47 patients had data available.

### 3.2. Short-Term and Long-Term Effectiveness of IIPT

**Figure 2:**
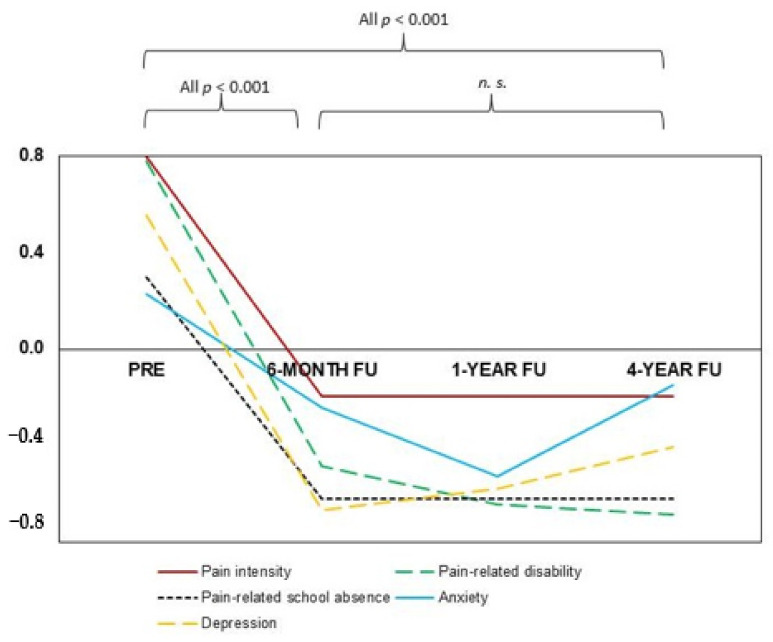
Z-standardized medians for the three pain-related and the two psychological measures at admission and the three follow-ups. The medians are standardized across all time points. *n.s.* = no significance (*p* > 0.05), ***p* < 0.001 = significant change**.

As shown in Table 2 and Figure 2, all pain-characteristics improved over time.

#### 3.2.1. Pain-Related Outcome Parameters

A repeated measures ANOVA determined that pain-related disability (F(3, 120) = 27.52, *p* < 0.001, partial η^2^ = 0.41) and pain intensity (F(3, 123) = 18.34, *p* < 0.001, partial η^2^ = 0.31) differed significantly between assessment time points. Post hoc tests using the Bonferroni correction revealed that both parameters were significantly reduced compared to PRE at 6-MONTH FOLLOW-UP (both *p* < 0.001), 1-YEAR FOLLOW-UP (both *p* < 0.001) as well as at 4-YEAR FOLLOW-UP (both *p* < 0.001). There were no significant changes in pain-related disability (all *p* = 1.000) or in pain intensity (6-MONTH to 1-YEAR: *p* = 1.000; 6-MONTH to 4-YEAR: *p* = 0.750; 1-YEAR to 4-YEAR: *p* = 1.000) between the follow-up time points.

Moreover, a repeated-measures ANOVA with Greenhouse-Geisser correction determined that missed school days (F(1.59, 50.71) = 28.26, *p* < 0.001, partial η^2^ = 0.47) differed significantly between assessment time points. Bonferroni-adjusted post hoc analyses detected that school absence was significantly reduced compared to PRE at 6-MONTH FOLLOW-UP (*p* < 0.001), 1-YEAR FOLLOW-UP (*p* < 0.001), as well as at 4-YEAR FOLLOW-UP (*p* < 0.001).

#### 3.2.2. Psychological Outcome Parameters

A repeated-measures ANOVA with Greenhouse-Geisser correction detected a significant difference between assessment time points for depression (F(1.99, 59.73) = 6.47, *p* = 0.003, partial η^2^ = 0.18). Bonferroni-corrected post hoc tests revealed a significant reduction from PRE to 6-MONTH FOLLOW-UP (*p* < 0.001) and from PRE to 1-YEAR FOLLOW-UP (*p* = 0.006), but not from PRE to 4-YEAR FOLLOW-UP (*p* = 0.077). There were no other significant changes in depression between the follow-ups (all *p* = 1.000).

Concurrently, a repeated-measures ANOVA showed a significant difference in anxiety between assessment time points (F(3, 84) = 5.11, *p* = 0.003, partial η^2^ = 0.15). Bonferroni-corrected post hoc tests revealed a significant reduction from PRE to 6-MONTH FOLLOW-UP (*p* = 0.001), but not from PRE to 1-YEAR FOLLOW-UP (*p* = 0.066) or from PRE to 4-YEAR FOLLOW-UP (*p* = 0.222). Again, there were no other significant changes in anxiety between the follow-ups (all *p* = 1.000).

## 4. Discussion

This longitudinal observational study aimed to investigate the short- and long-term effects of pediatric IIPT for patients with chronic headache. The six-month follow-up data demonstrated that short-term improvement was found in all pain-related and psychological parameters; patients suffered from less intense pain and less pain-related disability, while they attended school more frequently and indicated lower levels of depression and anxiety. Regarding the long-term effects of IIPT, there was a beneficial decline in pain-related outcome parameters; four years after IIPT, patients again reported less pain-related disability, pain intensity, and school absence. However, no significant long-term treatment effects of IIPT on anxiety and depression could be detected. Furthermore, the outcome parameters did not improve between the follow-ups, indicating that the effects of IIPT mostly kick in within the first year after treatment.

The findings of this study are in line with previous research on the effectiveness of pediatric IIPT. Shulman et al. [12] also found short-term effectiveness of IIPT for children and adolescents with chronic headache regarding pain intensity, pain-related disability, and school attendance. However, these findings should be interpreted carefully as the sample sizes of each follow-up differed substantially between the outcome parameters. Similar effects were found by Benore et al. [11], who showed that these beneficial effects of IIPT on pediatric chronic headache patients could be maintained up to one year after discharge. Additionally, at their 12-month follow-up, they detected improvements in pain-related outcome domains, as well as psychological parameters, such as depression and general anxiety, but not in general quality of life [11]. However, due to the very high drop-out rate of over 86% in this study, the risk of a systematic bias in the follow-up data is not unlikely.

Despite the positive findings regarding the short- and long-term treatment effects of IIPT on pain-related parameters in children and adolescents with chronic headache, the improvements in general anxiety and depression were not replicated in our study. Although data still suggests a remarkable decrease in general anxiety and depression levels from admission to four years after discharge, the trend for improvement was not significant over this period. This corresponds with findings in previous research, which found no long-term effects of IIPT on anxiety and depression in pediatric patients with various pain complaints [20]. One possible explanation for the short-lived beneficial effects on anxiety and depression might be the fact that IIPT, while certainly addressing aspects of emotion-related health issues and psychological comorbidities, it primarily focuses on the improvement of pain-specific outcome domains and daily functioning. In this regard, Thabrew et al. [21] detected in their review of psychological therapies for anxiety and depression in pediatric patients with long-term or chronic illnesses that psychological interventions specifically designed to reduce anxiety or depression were more effective at doing so than psychological therapies designed to reduce pain or to improve coping and social skills. However, Moessner et al. [22] detected a positive effect of an aftercare program following IIPT in a group of adult low back pain patients. Therefore, a specialized psychosocial aftercare program for pediatric chronic pain patients might be a way to not only consolidate but also further enhance pain-related and psychological outcome parameters up to six months after discharge. Such aftercare programs might have not only short-term but also long-term impacts on stabilizing the positive effects of IIPT.

There are several limitations to consider when interpreting these results and to attend to for future research. Even though the attrition rate after four years was an acceptable 33% and the drop-out analyses did not show any baseline differences between responders and drop-outs, systematic bias cannot be eliminated. Future research should therefore strongly focus on ways to increase participation adherence in longitudinal studies. Moreover, in this longitudinal observation study, a natural remission is not controlled for. Previous research indicated that a natural remission might especially be likely in males [23]. Furthermore, the differential effectiveness of the different treatment modules of IIPT for children and adolescents with chronic headache remains unclear. To further adjust IIPT and increase the program’s effectiveness and efficiency, these separate treatment modules require further investigation. Lastly, potential factors interfering with long-term treatment success, such as the adherence to therapy recommendations, were not registered. These potential factors and their effect on outcome parameters need to be considered in future studies with larger sample sizes.

## 5. Conclusions

The current longitudinal observation study found distinct evidence for the short- and long-term effectiveness of IIPT for children and adolescents with chronic headaches. This intensive and specified therapy approach positively affected pain-related outcomes in the short- and long-term, as well as psychological outcomes in the short-term. Future research is required to identify methods to consolidate the beneficial effects of IIPT on psychological outcome domains in the long-term.

## Figures and Tables

**Table 1:** Socio-demographic and pain characteristics at study inclusion.

	All	Participants 4-Year Follow-up	Dropouts 4-Year Follow-Up	*p*-Value
	*n* = 70	*n* = 47	*n* = 23	
Sex [*n* (%)]				0.096
Female	54 (77.1%)	39 (83%)	15 (65.2%)	
Male	16 (22.9%)	8 (17%)	8 (34.8%)	
Age [M (SD)]	14.5 (2.1)	14.5 (2.2)	14.5 (2.0)	0.914
Pain onset ^a^ [M (SD)]	32.3 (34.2)	27.7 (31.0)	41.6 (39.1)	0.112
Average pain intensity ^b^ [M (SD)]	6.5 (2.1)	6.7 (2.1)	6.0 (2.0)	0.149
Missed school days ^c^ [M (SD)]	8.2 (7.0)	8.6 (7.0)	7.6 (7.1)	0.572
Pain-related disability ^d^ [M (SD)]	36.8 (9.1)	37.5 (8.9)	35.4 (9.5)	0.364
General anxiety ^f^ [M (SD)]	53.2 (10.1)	54.0 (9.0)	51.5 (12.2)	0.344
Depression ^e^ [M (SD)]	55.4 (9.9)	55.7 (10.2)	54.7 (9.4)	0.702

^a^ In ^a^ in months, ^b^ In the past 7 days, ^c^ In the past 4 weeks, ^d^ assessed via the Pain-related disability inventory (PPDI; range 12–60), ^e^ assessed via the Depression Inventory for Children and Adolescents (DIKJ); *t* values (range: 0–100), ^f^ assessed via the Anxiety Questionnaire for Pupils (AFS); *t* values (range: 0–100).

**Table 2:** Pain-related and psychological outcomes (means and standard deviations).

	PRE	6-Month Follow-Up	1-Year Follow-Up	4-Year Follow-Up
	*n* = 70	*n* = 65	*n* = 58	*n* = 47
Pain characteristics				
Average pain intensity (last 7 days) ^a^	6.6 (2.2)	4.1 (3.2)	3.9 (3.0)	3.4 (3.1)
Missed school days (last 4 weeks)	8.2 (7.0)	0.8 (2.1)	1.1 (2.1)	2.1 (3.8)
Pain-related disability ^b^	37.9 (8.6)	22.3 (10.4)	22.8 (12.2)	22.8 (12.0)
Psychological characteristics				
General anxiety ^c^	55.1 (10.3)	48.0 (11.2)	49.3 (13.2)	50.5 (6.6)
Depression ^d^	53.7 (8.4)	45.3 (11.0)	46.2 (13.0)	48.2 (11.2)

^a^ NRS: ^a^ assessed via a 0–10 NRS, ^b^ Paediatric Pain Disability Index (P-PDI); Range: 12–60, ^c^ assessed via the Anxiety Questionnaire for Pupils (AFS); *t* values (range: 0–100), ^d^ assessed via the Depression Inventory for Children and Adolescents (DIKJ); *t* values (range: 0–100).

## Data Availability

Data are available upon request.
